# Catabolic cytokines disrupt the circadian clock and the expression of clock-controlled genes in cartilage via an NFкB-dependent pathway

**DOI:** 10.1016/j.joca.2015.02.020

**Published:** 2015-11

**Authors:** B. Guo, N. Yang, E. Borysiewicz, M. Dudek, J.L. Williams, J. Li, E.S. Maywood, A. Adamson, M.H. Hastings, J.F. Bateman, M.R.H. White, R.P. Boot-Handford, Q.J. Meng

**Affiliations:** † Faculty of Life Sciences, University of Manchester, A.V. Hill Building, Oxford Road, Manchester, M13 9PT, UK; ‡ Department of Chronopharmacology, School of Pharmacy, Taishan Medical University, Chang Cheng Road, Shandong, 271016, PR China; § MRC Laboratory of Molecular Biology, Neurobiology Division, Francis Crick Ave, Cambridge, CB2 0QH, UK; ‖ Murdoch Childrens Research Institute, Parkville, Victoria, 3052, Australia; ¶ Wellcome Trust Centre for Cell Matrix Research, University of Manchester, Oxford Road, Manchester, M13 9PT, UK

**Keywords:** Circadian clock, Cartilage, Cytokine, Inflammation, Osteoarthritis

## Abstract

**Objective:**

To define how the catabolic cytokines (Interleukin 1 (IL-1) and tumor necrosis factor alpha (TNFα)) affect the circadian clock mechanism and the expression of clock-controlled catabolic genes within cartilage, and to identify the downstream pathways linking the cytokines to the molecular clock within chondrocytes.

**Methods:**

*Ex vivo* cartilage explants were isolated from the *Cry1*-luc or PER2::LUC clock reporter mice. Clock gene dynamics were monitored in real-time by bioluminescence photon counting. Gene expression changes were studied by qRT-PCR. Functional luc assays were used to study the function of the core Clock/BMAL1 complex in SW-1353 cells. NFкB pathway inhibitor and fluorescence live-imaging of cartilage were performed to study the underlying mechanisms.

**Results:**

Exposure to IL-1β severely disrupted circadian gene expression rhythms in cartilage. This effect was reversed by an anti-inflammatory drug dexamethasone, but not by other clock synchronizing agents. Circadian disruption mediated by IL-1β was accompanied by disregulated expression of endogenous clock genes and clock-controlled catabolic pathways. Mechanistically, NFкB signalling was involved in the effect of IL-1β on the cartilage clock in part through functional interference with the core Clock/BMAL1 complex. In contrast, TNFα had little impact on the circadian rhythm and clock gene expression in cartilage.

**Conclusion:**

In our experimental system (young healthy mouse cartilage), we demonstrate that IL-1β (but not TNFα) abolishes circadian rhythms in *Cry1*-luc and PER2::LUC gene expression. These data implicate disruption of the chondrocyte clock as a novel aspect of the catabolic responses of cartilage to pro-inflammatory cytokines, and provide an additional mechanism for how chronic joint inflammation may contribute to osteoarthritis (OA).

## Introduction

The circadian (24 hourly) clocks in the brain and periphery direct key aspects of physiology through rhythmic control of tissue-specific sets of downstream genes^1^, ^2^. Circadian disruption (e.g., during ageing) contributes to various disease states^2^. Core clock genes build an auto-regulatory feedback loop, including activators (*Bmal1* and *Clock*), repressors (*Pers* and *Crys*) and output genes (e.g., *Rev-erbs, Dbp and Nfil3*)^1^, ^2^. Articular cartilage that covers the end of long bones comprises an abundant extracellular matrix (ECM) and a sparse population of chondrocytes which are essential for producing, assembling and turning over the ECM^3^. Progressive degeneration and destruction of articular cartilage is a common feature of various forms of arthritis, including rheumatoid arthritis (RA) and osteoarthritis (OA). We and other groups have previously reported functional circadian clocks in chondrocytes that control key molecules in the catabolic and anabolic pathways^4^, ^5^, ^6^. OA is the most prevalent joint disease and has a complex aetiology. Ageing, mechanical injury and obesity are among the major risk factors for OA^7^. We have demonstrated that the circadian oscillations in cartilage become damped during ageing, and that the expression of clock genes in cartilage changes during the initiation stage of OA development in an experimental mouse OA model^4^. Therefore, the disruption to cartilage rhythms could be an important contributing factor for the pathogenesis of OA^8^. It is currently unknown, however, how ageing and mechanical injuries disrupt cartilage clock rhythm.

Recent evidence strongly suggests that the chronic low grade inflammation that accumulates with age may drive the early degeneration during the development of OA, as well as in the late stage cartilage destruction^9^, ^10^, ^11^. In aged, injured and OA joints, levels of pro-inflammatory and catabolic cytokines, e.g., Interleukin 1 (IL-1) and tumor necrosis factor alpha (TNFα) in the synovial cavity are elevated in both animal models and humans, with signs of synovitis^9^, ^10^. These pro-inflammatory cytokines not only elicit catabolic effects (e.g., through induction of IL-6, Adamts4, MMPs which lead to matrix breakdown), but also suppress the anabolic pathways (such as Sox9 and Col2a1 expression), hence further impairing tissue repair^9^, ^10^, ^11^.

Application of IL-1β and TNFα alters clock gene expression in synovial fibroblasts from RA and OA patients^12^, ^13^. No studies have examined, however, how these catabolic cytokines impact on the clocks in cartilage. Given the circadian control of cartilage tissue homeostasis, we hypothesized that the build-up of pro-inflammatory cytokines may disrupt circadian rhythms in cartilage, which then compromises tissue homeostasis and predisposes cartilage to damage. We tested this hypothesis using cartilage tissue explants treated with pro-inflammatory stimuli and found that IL-1β profoundly disrupted circadian rhythms in the expression of clock genes. This effect involves interactions between NFкB signalling and the core clock mechanism. Interestingly, we also identified an unexpected lack of effect of TNFα in regulating circadian pathways and NFкB signalling in normal cartilage explant.

## Materials and methods

### Reagents and antibodies

IL-1β and TNFα were purchased from R&D, lipopolysaccharides (LPS), BMS-345541, dexamethasone (Dex), Forskolin (FSK) were purchased from Sigma. mClock and hBMAL1 expression plasmids were kind gifts from Dr. Kazuhiro Yagita (Kyoto Prefectural University of Medicine, Japan). *mAvp*-luc plasmid and its E-box mutant form, and luc assay were described previously^14^, ^15^. Plasmid transfections for SW-1353 cells were performed using Polyfect (Qiagen) according to the manufacturer's instructions.

### Animal maintenance

All animal experiments were conducted under the aegis of the 1986 Home Office Animal Procedures Act (UK) and following local ethical review. *Cry1*-luc mice were previously described^16^. In this transgenic mouse line, the endogenous *Cry1* promoter is fused with a luciferase reporter, allowing real-time monitoring of the *Cry1* gene dynamics by bioluminescence recording. PER2::LUC mice were kindly provided by Professor J. Takahashi (the University of Texas Southwestern Medical Center). In this knock-in mouse, endogenous PER2 protein is fused in-frame with a luciferase reporter^17^. 8–12 weeks old mice were maintained at 20–22°C, on standard rodent breeder or maintenance chow. Mice were entrained to a 12 h light and 12 h dark (LD) cycle.

### Cartilage explant cultures and bioluminescence recording

Cartilage cultures from clock reporter mice were prepared by dissection of the cartilaginous portion of the xiphoid process, or by dissection of the femoral head cartilage from 2 to 4 weeks old mice^4^. Cartilage was cultured on 0.4-μm cell culture inserts (Millipore), and bioluminescence was recorded in real time using a LumiCycle apparatus (Actimetrics). Baseline subtraction was carried out using a 24-h moving average. For cytokine treatment studies in cartilage tissue explants^18^, cartilage tissues were cultured under LumiCycle recording. After 2–3 days, cytokines were applied. For the effects of NFкB pathway inhibitors, tissues were pretreated with these drugs 15 min prior to cytokine treatment. For Dex and FSK, tissues were treated with cytokines first, immediately followed by Dex or FSK. The treatment agent was left continuously with the samples thereafter while the luminescence patterns were recorded for at least 7 days.

### SW-1353 cell culture

The SW-1353 chondrocyte-like cells^19^ used herein constitutively overexpress *Sox9* to drive *Col2a1* expression and maintain a chondrocyte-like phenotype. Cells were cultured in the following medium, DMEM with 4.5 g/L glucose, Glutamax and pyruvate, supplemented with 10% FBS, 100 U/mL Penicillin and 100 μg/mL streptomycin. Cultures were maintained at 37°C (5% CO_2_).

### Gene expression analysis

Ribonucleic acid (RNA) was extracted from cartilage explants or cultured SW-1353 cells using the Qiagen RNeasy purification system. cDNA was prepared using the Superscript II reverse transcriptase (Invitrogen) and analysed for gene expression using quantitative real-time PCR with TaqMan (Applied Biosystems) chemistry. Probeset was ordered (pre-validated) from Applied Biosystems and described previously^4^, ^15^. The fluorescence was recorded from cycle 2 with the linear phase being between cycles 12 and 34. Each sample was run in triplicate. The data were analysed using the 2^−ΔΔCT^ method. To calculate a relative change the average of the 3 replicate Ct values for each sample was used.

### Live tissue imaging

Articular or xiphoid cartilage tissue from p65-DsRed mouse was embedded in the Matrigel matrix (BD Biosciences) in 35-mm glass bottom Cellview dishes (Greiner Bio-one). Images were acquired with a Zeiss LSM 780 Confocal Inverted Microscope in a humidified CO_2_ incubator (at 37°C, 5% CO_2_) with a C-Apochromat 40×/1.2 W Korr objective. During imaging tissue was treated with IL-1β (5–20 ng/Ml) or TNFα (up to 40 ng/mL). DsRedXP tagged p65 was visualized by excitation with a green helium neon laser (543 nm) and detection through both a 545-nm dichroic mirror and a 560-nm long pass filter. Data capture was performed using ZEN2010B software (Zeiss).

### Statistical analysis

Data were evaluated using Student's *t* test. Results are presented as mean ± 95% confidence interval from at least three independent experiments.

## Results

### IL-1β but not TNFα disrupts the rhythmic expression of circadian clock genes in cartilage

**Fig. S1:**
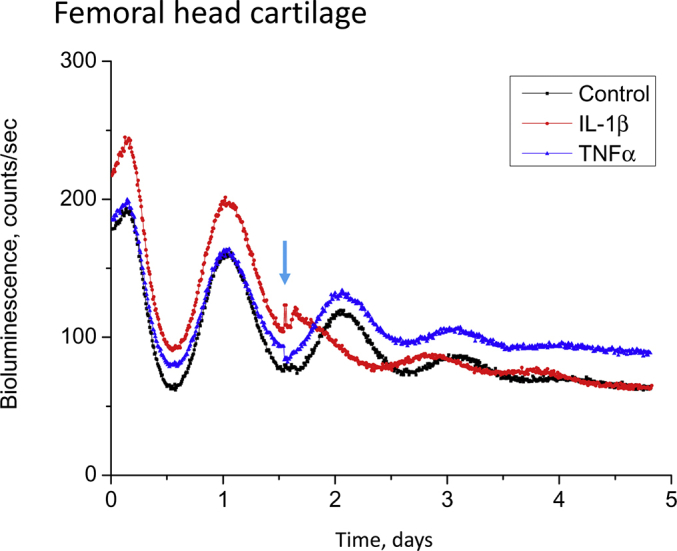
Representative bioluminescent traces from femoral head cartilage explants under real-time photon counting. The tissue explants were treated with IL-1β (5 ng/mL) or TNFα (10 ng/mL), as indicated by the arrow. Experiments were repeated three times (from three individual animals) with similar results.

We previously demonstrated robust circadian clocks in cartilages from the PER2::Luc fusion-protein reporter mouse^4^. Given the intimate links between the clock gene *Crys* and inflammation^20^, ^21^, we took advantage of the newly generated *Cry1*-luc^16^ circadian reporter mouse, which allowed us to track the dynamics of E-box-driven *Cry1* activity in *ex vivo* cartilage explants over 7–14 days. We have shown that cartilages from different anatomical locations (e.g., xiphoid, femoral head cartilage or knee cartilage) demonstrate little difference in their circadian oscillations^4^. Preliminary experiments also revealed similar responses of these different cartilages to cytokines (Fig. S1). Therefore, xiphoid cartilage tissue was used as a convenient and more amenable model in most of the subsequent studies.

**Fig. 1:**
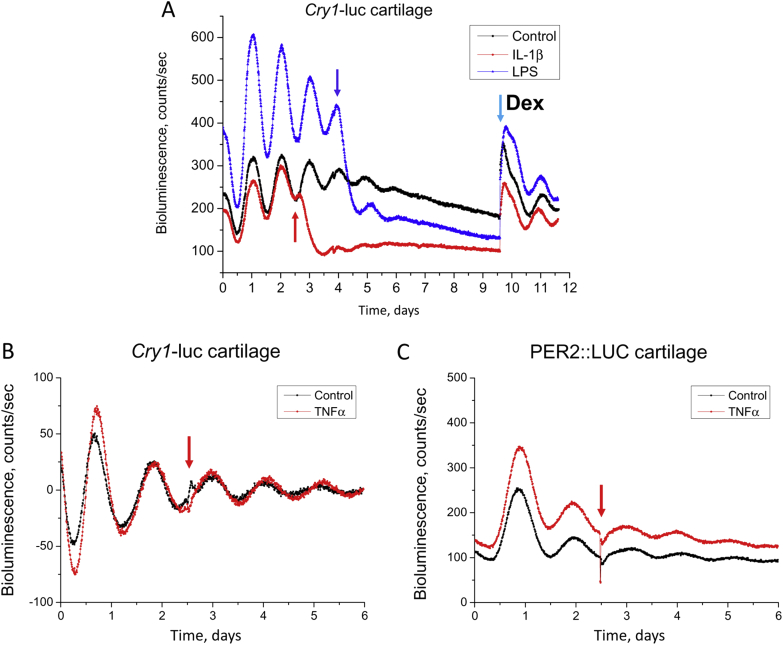
**IL-1β and LPS (but not TNFα) disrupt chondrocyte clock**. Representative *Cry1*-luc bioluminescent traces from xiphoid cartilage explants under real-time photon counting. (A) Circadian rhythm in *Cry1*-luc activity is abolished by IL-1β (5 ng/mL) and LPS (1 μg/mL). *N* = 4–6 animals per group. Red or blue arrow indicates time of treatment for IL-1β or LPS. Light blue arrow: application of Dexamethasone (Dex, 100 nM). (B, C) Treatment with TNFα (up to 20 ng/mL) has little effect on *Cry1*-luc or PER2::LUC oscillation in cartilage. Data were normalized to ease visualization. *N* = 6 animals per group.

Cartilage explants demonstrated robust circadian oscillations in *Cry1*-luc expression, with 24.3 ± 0.3 h period, indicating *Cry1* as a robust rhythmic gene in cartilage tissue [Fig. 1(A)]. To investigate a role of pro-inflammatory cytokines on cartilage clocks, we treated cartilage explants with IL-1β, lipopolysaccharides (LPS, a bacterial product that triggers strong inflammatory response), or TNFα. IL-1β and LPS treatments dampened circadian *Cry1*-luc bioluminescence rhythms, and this was associated with a constitutively lower expression level of *Cry1*-luc [Fig. 1(A)]. In contrast, treatment with TNFα had little effect on overall *Cry1*-luc expression or its circadian oscillation [Fig. 1(B)]. To investigate whether the lack of response to TNFα is specific for *Cry1*-luc gene dynamics, we treated cartilage from PER2::LUC mouse with TNFα. Here, similar lack of effect was observed [Fig. 1(C)], revealing a differential role of IL-1β and TNFα in cartilage, at least for the clock-related pathways.

### Cytokine-mediated disruption of the circadian clock in cartilage can be blocked by dexamethasone, but not by forskolin

**Fig. 2:**
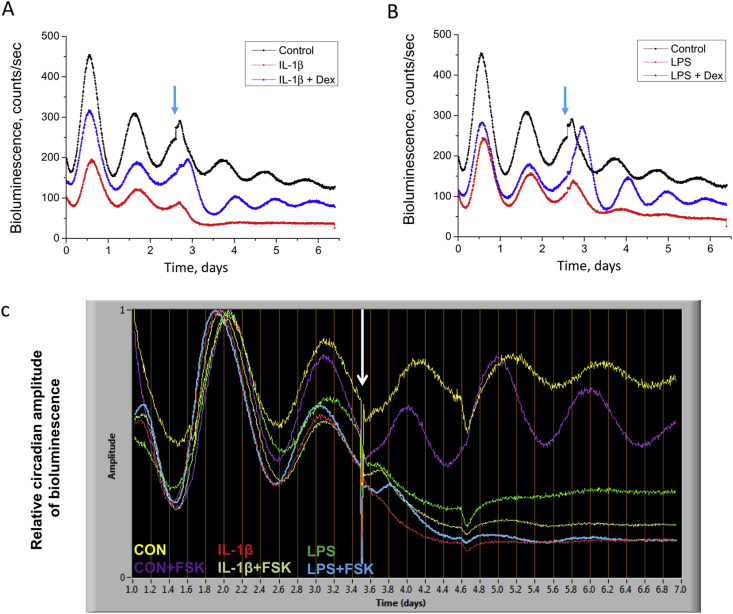
**Glucocorticoid, but not forskolin (FSK)**, **blocks the inflammatory disruption of the cartilage clock**. Representative *Cry1*-luc traces from xiphoid cartilage (*N* = 5 animals per group). Co-treatment with either Dex and IL-1β in (A), or Dex and LPS in (B), preserves circadian rhythm in *Cry1*-luc oscillation. Dex, 100 nM; IL-1β, 5 ng/mL; LPS, 1 μg/mL. (C) Co-treatments with FSK (10 μM) and IL-1β or FSK and LPS failed to synchronize the disrupted circadian rhythms in *Cry1*-luc oscillation. Data were normalized according to the amplitude before treatment to ease visualization. White arrow: time of treatment.

The disruption of circadian gene expression triggered by IL-1β or LPS was reversible because later application of dexamethasone, an anti-inflammatory agent, restored the overall level and oscillatory amplitude of the damped circadian rhythm [Fig. 1(A)]. Further, co-application of Dex with IL-1β or LPS effectively maintained the circadian oscillation of the cartilage explants [Fig. 2(A), (B)]. Interestingly, neither IL-1β nor LPS had any discernible effects on the circadian oscillations in tissue explants from lung or oesophagus (data not shown), suggesting a cartilage tissue-specific mechanism. These results reveal disruption of circadian clock in cartilage as a new target of the pro-inflammatory and catabolic cytokines.

The cytokine-induced disruption of circadian rhythmicity could be due to a population-level desynchronization of the constituent oscillatory cells within the tissue. Alternatively, it may reflect phase-independent molecular defects within the cell-autonomous clock oscillator. Given that Dex has both anti-inflammatory effects and clock-resetting actions in peripheral tissues, including cartilage^4^, ^22^, these alternative hypotheses could not be differentiated. To address whether desynchrony of cellular clocks might play a role in the damping of the rhythm, cartilages were treated with forskolin, a known clock-synchronizing agent. This increased the oscillatory amplitude of the control cartilage by ∼2.5 fold [Fig. 2(C)]. Co-application of either IL-1β or LPS with forskolin, however, failed to rescue the disrupted clocks [Fig. 2(C)], i.e., imposed synchronisation could not rescue the disruptive effect of the cytokines. These results indicate that a clock-desynchronizing effect is unlikely to be the main mechanism for the cytokine-mediated disruption of cartilage rhythm: rather a compromise of the cell-autonomous circadian mechanism is more likely.

### IL-1β-mediated disruption of the circadian clock in cartilage involves NFкB signalling

**Fig. S2:**
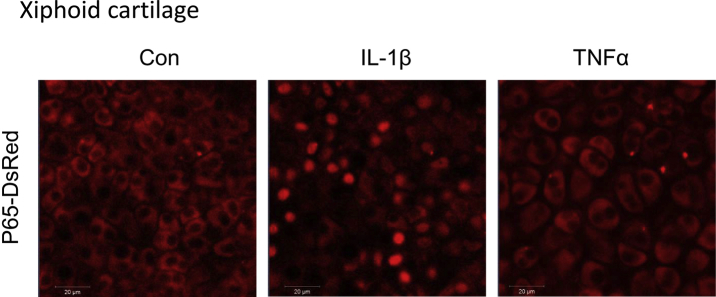
Differential effects of IL-1β and TNFα on the nuclear translocation of p65-DsRedXP in mouse xiphoid cartilage. *N* = 3 animals per group.

**Fig. 3:**
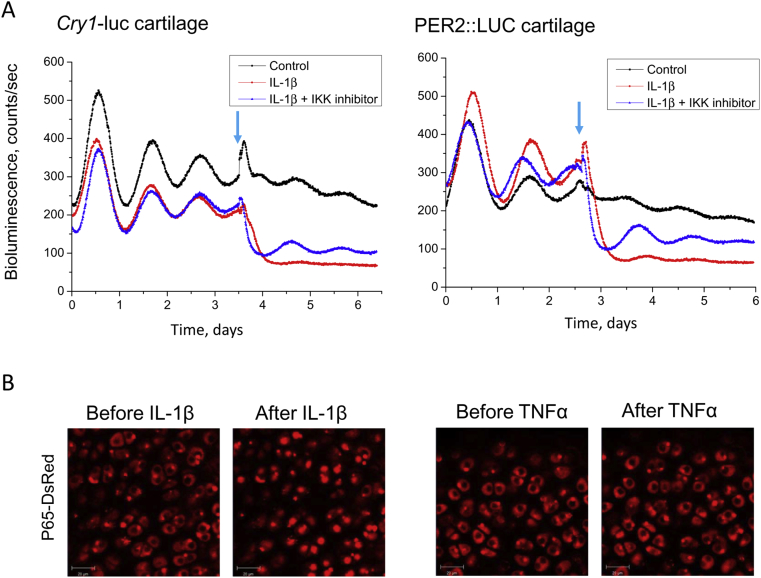
**NFкB signalling is involved in the IL-1β -mediated clock disruption in cartilage**. (A) Circadian rhythm in xiphoid cartilage explants from either the *Cry1*-luc or PER2::LUC reporter mice was abolished by IL-1β (5 ng/mL), but rescued by pre-treatment with an IKK1/2 inhibitor (BMS 345531, 10 μM). Representative result was shown, *N* = 4 animals per group. Arrow: time of treatment. (B) Differential effects of IL-1β and TNFα on the nuclear translocation of NFкB in mouse femoral head cartilage. Imaging of p65-DsRedXP expressing cartilage tissue was performed by confocal microscopy. Rapid nuclear translocation of p65-DsRed was observed within 40 min of IL-1β treatment (5–20 ng/mL). In contrast, treatment with TNFα at varying concentration (up to 40 ng/mL) and duration (up to 5 h) had little discernible effect. Representative data were shown, *N* = 3 animals per group.

One of the classic downstream pathways for pro-inflammatory cytokines is through NFкB signalling. Upon cytokine treatments, activation of IKK leads to phosphorylation and subsequent degradation of IкBα, thereby releasing NFкB, which then translocates to the nucleus to modulate its target genes^24^. Indeed, in the current study pre-treatment with the IKK1/2 inhibitor BMS-345541 prevented the loss of circadian rhythmicity in the IL-1β treated cartilage explants from both the *Cry1*-luc and the PER2::LUC reporter mice. It also partially restored the level of *Cry1*-luc or PER2::LUC expression, indicating the involvement of the NFкB pathway [Fig. 3(A)]. Finally, in young WT mouse femoral head or xiphoid cartilage tissues expressing the p65-DsRedXP protein fusion construct^25^, IL-1β treatment elicited rapid (within 40 min) nuclear translocation of p65, one of the main NFкB subunits [Fig. 3(B), Fig. S2]. In contrast, in cartilages treated with various doses of TNFα (up to 40 ng/mL), p65 remained largely cytoplasmic. These results favour the hypothesis that NFкB signalling is a mediator linking selective inflammatory signals to the dysregulation of the chondrocyte clock, and identified a differential response of cartilage NFкB signalling to IL-1β and TNFα.

### IL-1β simultaneously affects the clock genes and catabolic pathways

**Fig. S3:**
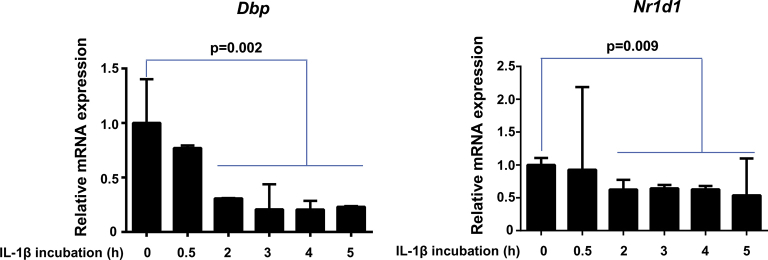
Time course for the reduction of E-box containing clock output genes (*Dbp and Nr1d1*) following IL-1β treatment in human chondrocyte-like SW-1353 cells.

**Fig. S4:**
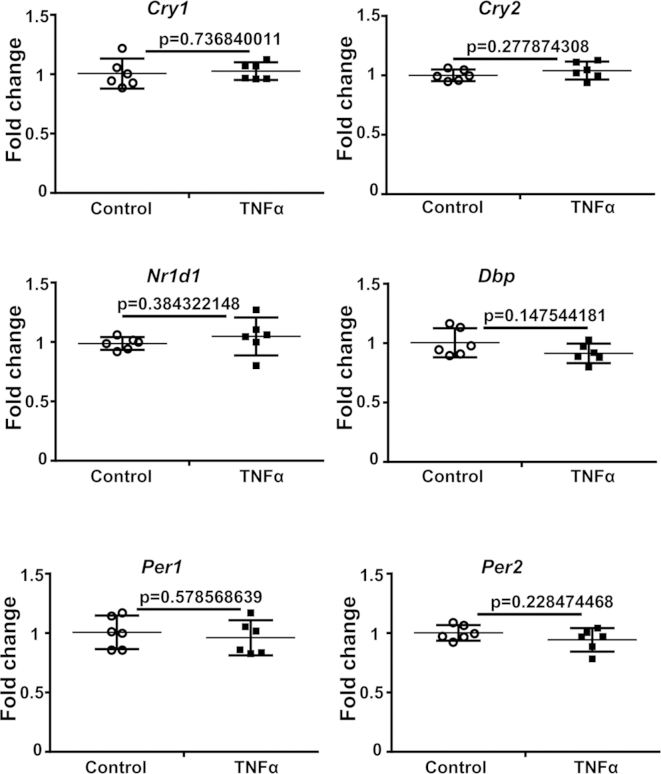
QRT-PCR showing lack of effect of TNFα (10 ng/mL) on the expression of E-box regulated clock genes in cartilage explants. *N* = 4 animals per group.

**Fig. 4:**
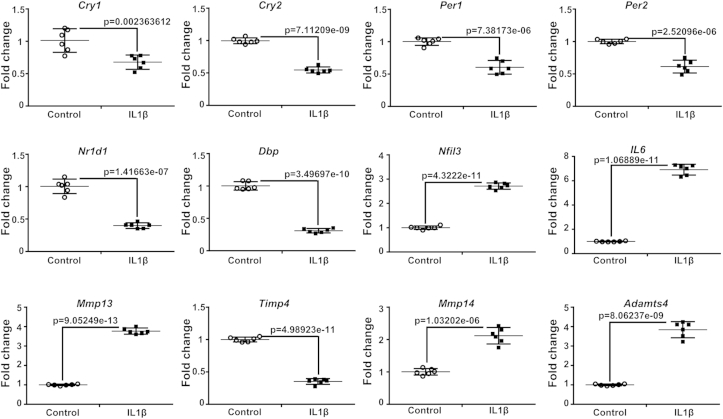
**Changes of clock genes and catabolic pathway-related genes upon IL-1β treatment in cartilage explants**. Cartilage explant cultures were synchronized by FSK (10 μM). Following clock synchronization, tissue explants were treated with IL-1β (5 ng/mL) 4 h before the corresponding peak of *Cry1*-luc expression (judged by bioluminescence recording of parallel *Cry1*-luc cartilages). Tissues were then removed at the relative peak of *Cry*1-luc activity for total RNA extraction. mRNA levels were quantified by Q-PCR. Data were normalized to *β-actin*. To calculate a relative change the average of the 3 replicate CT values for each sample was used. Mean ± 95% confidence interval; *t* test, *n* = 6 animals per group.

To test the alternative hypothesis, we investigated the effect of treatment with IL-1β on the expression of endogenous clock genes in cartilage explants. Consistent with the reduced *Cry1*-luc expression, IL-1β treatment (for 4 h) significantly reduced the *Cry1* and *Cry2* mRNA levels in cartilage (Fig. 4). *Cry* genes are positively regulated by the core Clock/BMAL1 complex through E-box *cis* elements in their promoters. Consistently, we found down-regulation of several other classic E-box regulated clock genes, including *Dbp, Nr1d1, Per1* and *Per2* upon IL-1β treatment (Fig. 4, Fig. S3). In contrast, TNFα had little effect on the expression of the above endogenous clock genes (Fig. S4). Interestingly, the expression of the clock gene *Nfil3* (with known functions in inflammation and negatively regulated by NR1D1^23^) showed significant up-regulation upon IL-1β treatment (Fig. 4). Of note is that *Dbp* and *Nfil3* are two clock output genes that have opposing roles in regulating downstream clock target genes that contain D-box *cis*-regulatory sequences. The opposite changes of these two clock outputs suggest a dysregulation of a spectrum of D-box containing cartilage genes.

We have previously shown that several catabolic pathway related genes in cartilage are clock-controlled^4^, such as *Mmp14* (a membrane-associated proteinase), *Timp4* (tissue inhibitor of MMP, member 4), and *Adamts4* (a major cytokine-inducible aggrecanase). Consistent with the changes of clock genes, IL-1β treatment significantly increased *Mmp14* and *Adamts4* expression, while suppressing *Timp4* levels [Fig. 4, (*P*) < 0.01 for all three genes]. These results suggest the possibility that changes of clock genes may be involved in linking cytokine signalling to the catabolic pathways within cartilage. As a control, we also measured two catabolic genes MMP13 and IL-6, which are well established targets of NFкB. Consistent with the potent nuclear translocation of p65 induced by IL-1β, significant increase of MMP13 and IL-6 expression was observed (Fig. 4).

### IL-1β treatment interferes with the function of the core clock complex

**Fig. 5:**
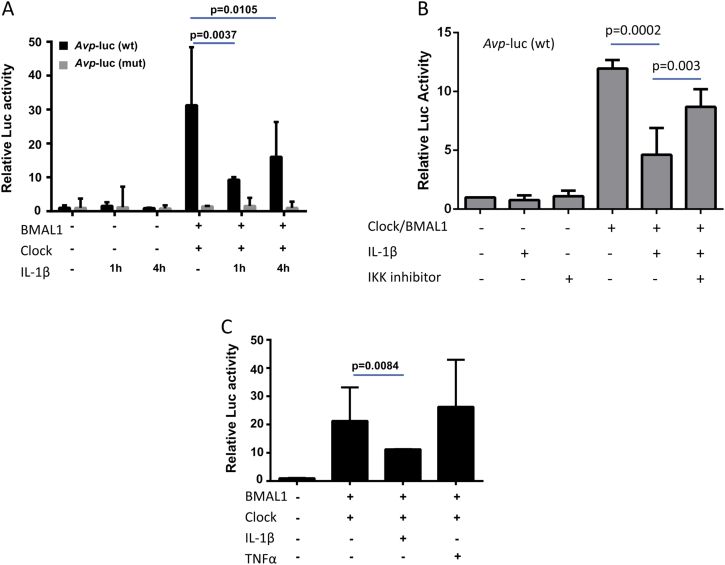
**IL-1β supresses the Clock/BMAL1 activation of E-box element in an NFкB-dependent manner in human chondrocyte-like cells**. Representative luc assay results in SW-1353 cells. Data (mean ± 95% confidence interval) was normalised to β-galactosidase reporter gene and expressed relative to control. *t* test, *N* = 4 culture replicates per experiment. Each experiment was repeated at least three times.(A, B) Expression plasmids for Clock and BMAL1 were co-expressed with *Avp*-luc promoter (wt or E-box mutant form) in SW-1353 cells. Cells were then treated with IL-1β (5 ng/mL for 1 or 4 h), with or without pretreatment with BMS 345531 (10 μM). Following cytokine incubation, cells were lysed for luc assay. (C) As above, the effect of TNFα (10 ng/mL for 4 h) on the Clock/BMAL1 transactivation of wt *Avp*-luc promoter was evaluated.

The down-regulation of E-box controlled clock genes suggests that IL-1β may disrupt the function of the core clock mechanism, i.e., the Clock/BMAL1-mediated transactivation of E-box elements. To dissect the molecular mechanism of the cytokine-mediated circadian disruption further, we performed functional promoter-luc assays in a SW-1353 chondrocyte-like cell line using a well-established E-box-containing *Avp* gene promoter-luc construct^14^. Co-expression of Clock and BMAL1 significantly increased the wild-type *Avp*-luc activity, but not that of the E-box mutated control plasmid. In SW-1353 cells treated with IL-1β, the ability of Clock/BMAL1 to transactivate the E-box promoter was significantly reduced [*P* < 0.01 at 1 h and *P* < 0.05 at 4 h, Fig. 5(A)]. Pre-treatment with the IKK1/2 inhibitor BMS-345541 partially blocked the suppressive effect of IL-1β on E-box activation, supporting a role of NFкB signalling in mediating the dampened clock function upon IL-1β treatment [Fig. 5(B)]. In contrast, treatment with TNFα had little effect on E-box transactivation [Fig. 5(C), (*P*) > 0.05], consistent with a lack of effect of this cytokine on clock gene oscillation. These results suggest that inflammatory cytokines may have cell-type specific roles in regulating their downstream targets and that IL-1β interferes with canonical circadian transcriptional mechanisms.

## Discussion

Our findings using cartilage tissue explants from the clock reporter mouse models identify the disruption of the circadian clock in cartilage as a novel target for joint inflammation. Given the diverse pathways controlled by the cartilage circadian clock, the profound disruption of clock genes may be involved in driving key aspects of the catabolic response of cartilage to chronic joint inflammation.

Cross-talk between inflammation/immune response and the circadian system has been reported in other tissues^26^, ^27^, ^28^, ^29^. For example, in macrophages and synovial fibroblasts, the Clock/NFкB interaction impedes the formation of the Clock/BMAL1 complex^26^. Consequently, activation of NFкB signalling leads to suppression of clock function in those cell types. To the best of our knowledge, this study is the first to demonstrate an unambiguous role of inflammation to circadian rhythms in clock gene expression in cartilage tissue. Clock disruption in synovial tissue/cells has been reported in arthritic diseases^12^, ^13^. Although, in those studies, the low resolution sampling and the absence of healthy tissue samples for comparison render the conclusions tentative. In contrast, our study using long-term real-time monitoring of clock gene dynamics provides *ex vivo* evidence that the cartilage tissue clock is a sensitive target for inflammatory cytokines.

**Fig. S5:**
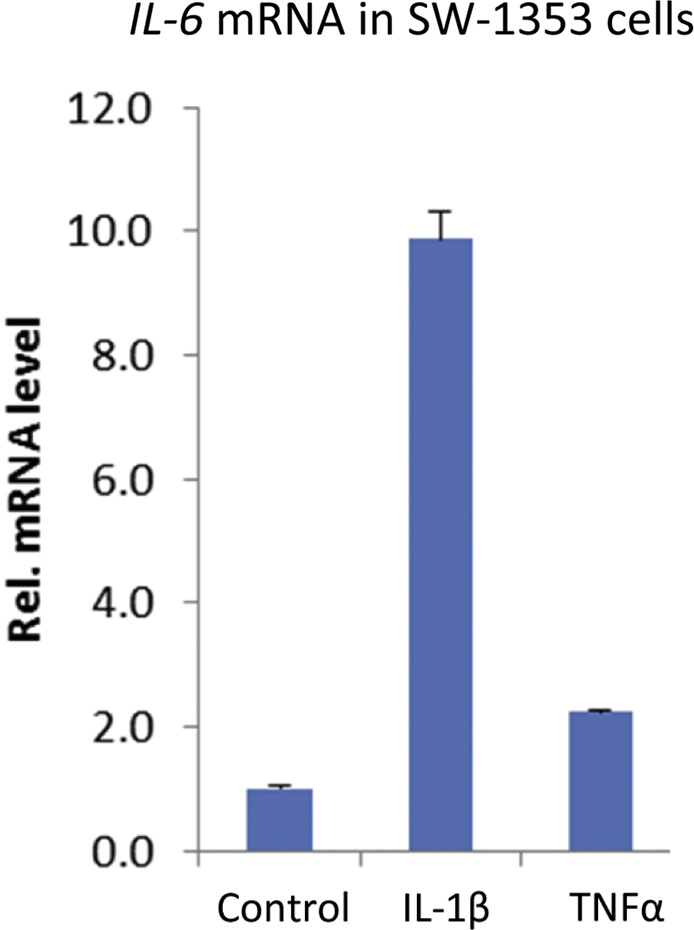
QRT-PCR reveals differential effects of IL-1β and TNFα on the induction of IL-6 mRNA in SW-1353 cells. *N* = 4 culture replicates per group.

**Fig. S6:**
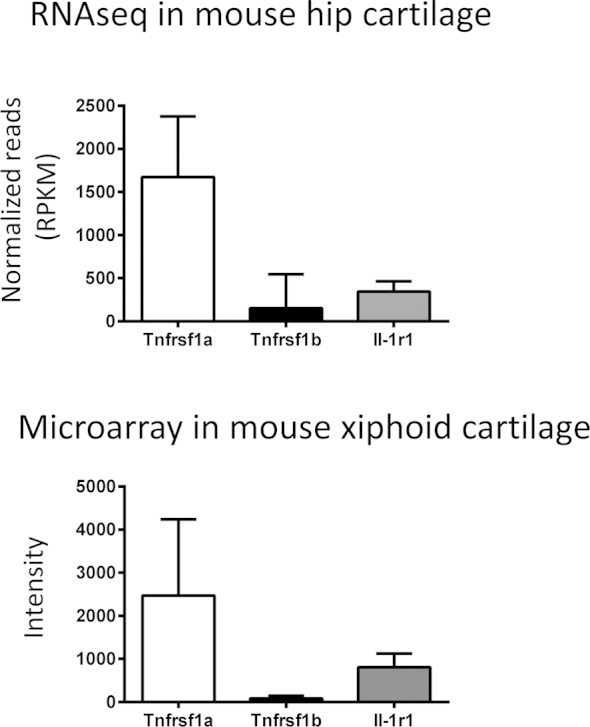
RNAseq and microarray results for the transcript levels of TNFrsf1a, TNFrsf1b and IL-1R1 in the hip and xiphoid cartilages of young adult (8–10 weeks old) WT mice. *N* = 4 animals for the hip RNAseq data; *N* = 2 samples (each sample was pooled from 5 animals) for the xihpoid microarray data. Data was expressed as mean ± 95% confidence interval.

We also identified a differential response of cartilage clocks to IL-1β and TNFα under normal (non-arthritic) conditions. It is likely that within the joint tissue, IL-1 has a predominant role in disruption of the cartilage clock and thus on matrix breakdown, while TNFα targets clocks in other cell types, such as the synovial cells or immune cells, as reported before^26^, ^29^. The finding that TNFα is not capable of driving NFкB nuclear translocation in normal cartilage is intriguing. Both IL-1 and TNFα have well established roles in both RA and OA^9^, ^10^, ^11^. The downstream signalling cascades and targets within cartilage tissue and chondrocytes are, however, likely to diverge. For example, microarray studies in SW-1353 cells revealed distinct target gene clusters in response to IL-1 or TNFα^19^. Catabolic genes like IL-6, BMP-2, MMP13 and COX-2 only respond to IL-1, with significantly lower or no response to TNFα^19^, ^30^. We were able to replicate the differential effects of IL-1 and TNFα on human IL-6 induction in SW-1353 cells (Fig. S5). Of note is that mouse and human chondrocytes are known to express receptors for TNFα^31^, ^32^. Consistent with previous reports, our own RNAseq results in mouse hip cartilage (unpublished) and microarray results in mouse xiphoid cartilage^4^ reveal that the transcript levels for TNFR1(TNFrsf1a) and IL-1R1 are readily detectible (Fig. S6). However, cytokine receptors are known to be differentially regulated during OA^33^. Therefore, it is not unlikely that arthritic chondrocytes become sensitive to TNFα. Moreover, the elevated levels of both TNFα and IL-1 in OA joints may have synergizing effects. Further investigation is clearly needed to assess how the circadian clocks in OA chondrocytes respond to these catabolic cytokines.

The novel concept of disruption of the circadian clock of cartilage as a new target of joint inflammation is significant because currently there is no disease-modifying therapy available for OA. Various anti-inflammation strategies have been proposed for OA, some of which (e.g., IL-1Ra therapies) are in clinical trials/use for limiting degradation of cartilage in RA and OA. So far, these traditional anti-inflammatory drugs are generally less effective in OA than in RA, and less well tolerated in the elderly OA patients. Our findings may provide new routes to combat chronic joint inflammation in OA patients, e.g., targeting the circadian clock itself by clock acting compounds^34^, ^35^, ^36^, which could be combined with the conventional anti-inflammatory drugs to reduce their dosage and, hence, their adverse effects.

In conclusion, we have demonstrated that IL-1β, but not TNFα, profoundly disrupts clock gene rhythms in *ex vivo* cartilage explants, and this effect involves NFкB signalling. Our data highlight disruption of the chondrocyte clock as a novel target for the responses of cartilage to pro-inflammatory/catabolic cytokines, and provide an additional mechanism for how chronic joint inflammation contributes to OA.

## Author contributions

Conception and design: QJM; Data acquisition: BQG, MD, EB, NY, JW, JL, ESM, AA; Data analysis and interpretation: BQG, MD, EB, NY, JL, ESM, MHH, AA, JFB, MRHW, RPBH, QJM; Drafting of the article: QJM, BQG, MHH, RPBH; Final approval of the article: all co-authors; Obtained funding: QJM, RPBH, JL, MRHW; Coordination and responsibility for the integrity of the work: QJM.

## Competing interest statement

All authors declare no competing interests relevant to this manuscript.
